# New York Letter

**Published:** 1896-03

**Authors:** Ogden C. Ludlow

**Affiliations:** 2309 Seventh Avenue; New York


### NEW YORK LETTER.

NEW YORK, February 15, 1896.

This being the season of the year in which cases of pneumonia, pleurisy, and empyema are very frequent, it seems opportune to refer to a recent discussion in the Academy of Medicine on empyema as it is found in young children. In discussing the bacteriology of this subject, Dr. Henry Koplik divided the cases, according to the mode of infection, into (1) metapneumonic; (2) streptococcus empyemas; (3) tubercular empymas, and (4) fetid empyemas. Fully two-thirds of the pleurisies occurring in young children have been estimated to be of the first variety. As bearing on the matter of diagnosis, Dr. August Caillé said that in small children the change in level of the fluid due to changes in the position of the patient was not easily detected. Several of the speakers agreed as to the very great difficulty experienced in actual practice in differentiating between a fluid effusion in the pleural cavity and pneumonia in infants under eighteen months old. The importance of that peculiar sense of resistance imparted to the finger used as a pleximeter, during percussion over fluid in a child's chest, was emphasized and dwelt upon by both Dr. Caillé and Dr. Winters, and the latter seemed to think that much of the difficulty in making the diagnosis of pleuritic effusion in these little ones would vanish if the physician took the precaution to see that the child made very deep inspirations while he was practicing auscultation. If this were done, a sound would be heard, which, although difficult to describe, is totally different from that heard over a pneumonic lung. All agreed that the crucial diagnostic test was the insertion of an aspirating needle, and Dr. Caillé made an important practical point by calling attention to the liability of getting into error by mistaking for pus the admixture of a few drops of serous fluid with some of the antiseptic solution left in the syringe. He advised covering the site of the puncture with a bit of gutta-percha tissue, made adhesive by moistening it with chloroform. Dr. W. P. Northrup insisted very strongly on the absolute necessity of using a needle of much larger caliber than that commonly employed, otherwise the failure to withdraw fluid could not be considered as positively indicating that no fluid was present. It is also important to make more than one puncture, should no fluid be found at the first attempt. There is no danger of injuring the liver if the puncture is not made on the right side below the eighth intercostal space. The sixth intercostal space is ordinarily a convenient point to select for exploratory puncture, but experience has shown that no matter where the opening be made for drainage, whether high up or low down, drainage is effective. From the number of cases following pneumonia it is evident that we must ever be on the alert in attending little children with pneumonia, to detect promptly the occurrence of an effusion, whether serous or purulent. It is the failure to draw the line sharply between the original pneumonia and the subsequent empyema that is responsible for so many deaths from infantile empyma. A safe rule is to make an exploratory puncture in cases where the protracted course of the pneumonia leads us to suspect that we may be dealing with this very important and common sequela. Dr. H. D. Chapin says that if, in the course of a pneumonia, we observe an area of dullness beginning at the apex, there is good reason for believing that it is due to a pleuritic effusion.

In the treatment of infantile empyema, Dr. Joseph E. Winters said that we should not forget that an empyema does not heal up from the bottom, but by expansion of the lung and the ascent of the diaphragm. If there were no cyanosis or other special contraindication, he did not object to placing the patient *partially* under the influence of an anesthetic, preferably chloroform, but full anesthesia was very undesirable, as it was by the coughing and crying, aided by the expansion of the lung, that we expected to secure the expulsion of the pus from the chest. For this reason it was also useful, just after making the incision into the chest, to give the child a teaspoonful of undiluted whisky. The incision should be at least two inches in length, and two or three inches above the normal base of the thoracic cavity. The patient should lie on the affected side and close to the edge of the table. Dr. Winters is very strongly opposed to resection of a rib in these cases of empyema in little children, (1) because he considers it entirely unnecessary; and (2) because the mortality following rib resection is 80 per cent. as compared with a mortality of 25 per cent. after simple incision. On the other hand, Dr. B. Scharlau says, from an experience of over one hundred operations for resection of rib for empyema, that he is convinced that convalescence is much more rapid than after incision without resection of a rib; and in this opinion he is supported by Dr. Joseph D. Bryant, the president of the academy. Irrigation of the pleural cavity after incision is not very commonly practiced here, although Dr. Winters advocates the use of hot water for this purpose, on the ground that it favors the escape of large coagula. This advantage is offset, in Dr. Caillé's opinion, by the effect of irrigation in breaking up adhesions between the lung and the wall of the thorax.

Tuberculosis is ever with us, and it is right that the laity as well as the medical profession should be freely informed regarding such acquisitions to our knowledge as bear upon the dangers of disseminating this disease, and the best means of preventing this; but so much has appeared in newspapers and other journals in recent years, that the public begins to show evidence at times of being a little "panicky." This has led to many heartless and unnatural acts, such as forsaking dear ones suffering from this malady, or placing them in some institution, and endeavoring to keep them from returning home for fear of others in the family contracting the disease. This is another exemplification of the old adage, "A little knowledge is dangerous." It is fortunate, therefore, that there has been a recent contribution to medical literature which dispels some of the groundless fears entertained by many, both within and without the profession, and makes our knowledge more definite. Dr. Irwin H. Hance, who has spent several years with Dr. Trudeau, in the Adirondack cottage sanitarium for consumptives, has presented to the Academy here the results of a study he has made regarding the infectiousness of the dust obtained from the various parts of this sanitarium. The experience of that well known sanitarium has lead to two most important conclusions, viz.: (1) that buildings in which consumptives are housed do not readily become infected with the germs of tuberculosis, if certain simple precautions be taken; and (2) that a tuberculous patient does not infect others by contact, but by their secretions, chiefly the sputum. The dried and pulverized sputum may be considered in practice as the infectious element. It has been found that dried sputum will retain its vitality for at least three years, yet the bacteriological experiments of Dr. Hance have shown that sixteen out of seventeen buildings that had been inhabited by consumptives for upward of ten years were absolutely free from tubercular infectious material. The patients here are taught the danger to themselves as well as to others from neglecting to take care of their sputa properly. They expectorate into paper cuspidores or into Japanese paper napkins, which are afterwards destroyed by fire. Nothing into which the patient expectorates is used a second time, and the sputum is not allowed to become dry. Sublimate solutions are not proper for the disinfection of masses of sputum. The sputum should be mixed with an equal quantity of a five per cent. solution of carbolic acid. It is very reassuring to know that no patient who has been admitted to this sanitarium with lung disease but without tubercle bacilli having been found, has developed tuberculosis in the sanitarium, and also that not one of the twenty-five attendants have developed the disease in the sanitarium. Of course, an abundance of sunlight and fresh air are considered as most important factors in connection with the management of a sanitarium of this kind. The practice in many country places of keeping the houses darkened most of the time, and but rarely ventilated, Dr. Andrew H. Smith has done well in calling attention to, for, as he very properly says, there are instances on record in which many members of a family have been swept away by consumption under such circumstances as to point very strongly to this kind of house infection, rather than to heredity, as the cause. Quite recently, it is said, some enthusiastic bacteriologist, in his ardent search after "bugs," forgot his reverence for the past, and carried on his quest among some tapestries, much-esteemed heirlooms, that had been hanging in a house for several generations. Fancy his delight, and the horror of the non-scientific portion of the world, when these valued relics of the past were found teeming with the germs of tuberculosis! This reminds me of what Dr. A. Jacobi said about the lowest tenement-houses often not being as unhealthy as the more pretentious cheap flat-houses, for the reason that the halls and stairs of the former were uncovered, while in the latter they were covered with dirty carpet, full of filth and disease.

In a recent discussion before the Surgical Section, the matter of cocaine anesthesia was incidentally touched upon. Dr. J. A. Wyeth said that he had performed many hundred operations under cocaine, and with the exception of a few urethral cases which he had operated upon before he had learned how to use the cocaine, he had never seen any toxic effects from it. His plan was to use a four per cent. solution of cocaine, and to introduce not more than two minims at any one place. As this part was quickly incised, most of the cocaine solution flowed out after such short contact with the tissues that there was no time for the production of constitutional symptoms. He had done most of the common surgical operations under cocaine anesthesia, including operations for appendicitis, and the ligation of important arteries, and he had rarely found it necessary to use more than twenty or twenty-five minims of a four per cent. solution. Dr. B. Farquhar Curtis said that he had done gastrotomy and laparotomy satisfactorily under cocaine. Dr. Willy Meyer said that he did not feel that it was quite safe to use cocaine except when combined with nitroglycerine; Dr. Manley and Dr. Dawbarn preferred to combat the bad effect of the cocaine, in contracting the cerebral vessels, by giving at the same time, an alcoholic stimulant, which also had the advantage of intensfying the analgesic action. Dr. Manley said he had performed major amputations under cocaine with the greatest satisfaction, but he preferred to use a weak solution, and not to exceed a total quantity of one grain.

That somewhat old and worn-out theme, for discussion—"appendicitis"—which continues to bob up as persistently as does antitoxin, was the chief subject for discussion before the Surgical Section the other evening. Dr. Beverly Robinson infused an element of novelty into it by propounding a theory as to its etiology which, if correct, is bound to deprive the surgeons of many operations. He argues that recent anatomical investigations have proved that there is much lymphoid tissue in the vermiform appendix, and that the structure is therefore not unlike that of the tonsils of the throat. It is well known that the latter are very frequently the seat of acute or subacute inflammation, in which an underlying rheumatic element figures quite prominently. It is on this account that the salicylates and guaiacum, and like remedies have gained a reputation in the treatment of tonsillitis. Now, Dr. Robinson proposes to extend this line of therapeutics still further, and apply it to the subacute inflammations of the appendix vermiformis. He has become thoroughly convinced that foreign bodies in the appendix are entirely too rare to account for the very large number of cases of appendicitis, and his own experience has led him to believe that medical men need no longer say in tones of despair that they have "no treatment for appendicitis." In this connection it is interesting to note that Dr. A. E. Gallant, in examining about two hundred appendices for foreign bodies, only found seeds in a single case. There were fecal concretions in other cases, but not true foreign bodies. It should not be inferred from the foregoing that Dr. Robinson claims that by the administration of anti-rheumatic remedies in most cases of appendicitis, all need for surgical interference will disappear, but simply that this appears to be a rational therapeutic procedure, calculated to relieve and cure many cases of appendicitis, so that many of them will recover without resort to operation, as they used to do before we ever heard of "appendicitis."

OGDEN C. LUDLOW, M.D.

2309 *Seventh Avenue.*

---

I have been led to feel that when the symptoms indicate appendicitis, the combination of a rapid pulse and respiration, going with a temperature normal, or nearly so, points to the necessity of prompt operation.—DAWBARN, *Medical Record.*

